# MiR-219-5p inhibits growth and metastasis of ovarian cancer cells by targeting HMGA2

**DOI:** 10.1186/s40659-018-0199-y

**Published:** 2018-11-24

**Authors:** Feng Xing, Zhijiao Song, Yuanying He

**Affiliations:** Department of Obstetrics and Gynecology, Shanghai Tenth People’s Hospital of Tongji University, Tongji University School of Medicine, No 301 Middle Yan Chang Road, Shanghai, 200072 China

**Keywords:** Ovarian cancer, miR-219-5p, HMGA2, Growth, Metastasis

## Abstract

**Background:**

Accumulating studies have demonstrated that high-mobility group A2 (HMGA2), an oncofetal protein, plays a role in tumor development and progression. However, the molecular role of HMGA2 in ovarian carcinoma is yet to be established. MicroRNAs (miRNAs), a group of small noncoding RNAs, negatively regulate gene expression and their dysregulation has been implicated in tumorigenesis. The aim of this study was to investigate the potential involvement of a specific miRNA, miR-219-5p, in HMGA2-induced ovarian cancer.

**Methods:**

The ovarian cancer cell line, SKOV3, was employed, and miR-219-5p and HMGA2 overexpression vectors constructed. The CCK-8 kit was used to determine cell proliferation and the Transwell^®^ assay used to measure cell invasion and migration. RT-PCR and western blot analyses were applied to analyze the expression of miR-219-5p and HMGA2, and the luciferase reporter assay used to examine the interactions between miR-219-5p and HMGA2. Nude mice were employed to characterize in vivo tumor growth regulation.

**Results:**

Expression of miR-219-5p led to suppression of proliferation, invasion and migration of the ovarian cancer cell line, SKOV3, by targeting HMGA2. The inhibitory effects of miR-219-5p were reversed upon overexpression of HMGA2. Data from the luciferase reporter assay showed that miR-219-5p downregulates HMGA2 via direct integration with its 3′-UTR. Consistent with in vitro findings, expression of miR-219-5p led to significant inhibition of tumor growth in vivo.

**Conclusion:**

Our results collectively suggest that miR-219-5p inhibits tumor growth and metastasis by targeting HMGA2.

## Background

Ovarian cancer has the highest mortality rate of all gynecologic neoplasms and is the fifth leading cause of cancer-related death in females in Western countries [1, 2]. Although remarkable advances have been made in the treatment of this tumor type [3], the long-term prognosis of metastatic melanoma remains poor.

High-mobility group A2 (HMGA2) is a small nuclear protein belonging to the high mobility group (HMG) family. The protein modulates multiple genes through effects on protein-DNA or protein–protein interactions [4, 5]. HMGA2 has been identified as an oncoprotein that is frequently upregulated in a variety of cancers, including breast cancer [6], esophageal squamous carcinoma [7], colorectal cancer [8, 9], ovarian tumors [10]. However, the mechanisms underlying regulation of HMGA2 in ovarian tumors are currently unclear.

MicroRNAs (miRNAs) are small noncoding RNAs 15–22-nucleotides in length [11] proposed to be correlated with tumor growth and metastasis based on their protumor or antitumor effects in a number of neoplasms [12]. Recent bioinformatics analyses (http://www.genecards.org/) have revealed that miR-219-5p targets the 3′UTR of HMGA2. Abnormal expression and tumor suppressor activity of miR-219-5p in different malignant cancer types have been documented in the literature [13, 14]. However, the role of miR-219-5p in ovarian cancer remains unclear at present.

The main aim of this investigation was to establish whether miR-219-5p-HMGA2 interactions play a regulatory role in ovarian cancer progression. Our results showed that increased expression of miR-219-5p leads to significant reversal of HMGA2-induced cell migration, invasion and proliferation of ovarian cancer cells. In addition, miR-219-5p was determined as a regulator that inhibits HMGA2 expression in ovarian cancer.

## Materials and methods

### Ethics statement

All animal experiments were approved by the Ethics Committee of Shanghai Tenth People’s Hospital, Shanghai, China. Surgical procedures were performed under anesthesia and every effort was made to minimize suffering of animals. All mice were anesthetized via intraperitoneal injection of sodium pentobarbital (30 mg/kg).

### Cell lines and cultures

Both SKOV3 and HEK293T cell lines were obtained from the American Type Culture Collection (Manassas, VA, USA). SKOV3 cells were cultured in RPMI 1640 (Invitrogen, California, USA), and HEK293T in Dulbecco’s Modified Eagle Medium (Invitrogen, California, USA) supplemented with 10% fetal bovine serum (FBS; Invitrogen, California, USA) at 37 °C in 5% CO_2_.

### Transfection of cells with miR-219-5p mimic or HMGA2 overexpression vector

The miR-219-5p mimic vector was synthesized by GenePharma (Shanghai, China). Full-length HMGA2 from the human cDNA library was cloned into pCDNA3.1 vector. Cells were transfected using the Lipofectamine 2000 reagent (Thermo Scientific, Massachusetts, USA) according to manufacturer’s instructions.

### Qualitative PCR analysis

Total RNA was extracted from tissues and cells using TRIzol reagent (Invitrogen, California, USA) in keeping with the reagent kit protocol and cDNA amplified using the TaqMan miRNA reverse transcription kit (Invitrogen, California, USA). HMGA2, miR-219-5p and U6 mRNA levels were determined via qPCR using the TaqMan human miRNA assay kit. Relative fold difference was measured using the 2^−∆∆CT^ method. The following primers were employed: HMGA2 (F) 5′-CTCAAAAGAAAGCAGAAGCCACTG-3′ and (R) 5′-TGAGCAGGCTTCTTCTGAACAACT-3′; miR-219-5p (F) 5′-ACACTCCAGCTGGGTGATTGTCCAAACGCAAT-3′ and (R) 5′-CTCAACTGGTGTCGTGGA-3′; U6 (F) 5′-CTCGCTTCGGCAGCACA-3′ and (R) 5′-AACGCTTCACGAATTTGCGT-3′.

### CCK-8 assay

Cell proliferation was detected with the CCK-8 assay according to the manufacturer’s protocol (Invitrogen, California, USA). SKOV3 cells from different groups were cultured in 96-well plates under the same conditions. After 0, 24, 48 and 72 h, a mixture of 90 μl fresh culture medium and 10 μl CCK-8 solution was added to wells. Following further incubation of SKOV3 cells at 37 °C for 2 h, cells were examined using a microplate reader at a wavelength of 450 nm.

### Luciferase reporter assay

HMGA2 with wild-type or mutant 3′-UTR was generated and cloned into the firefly luciferase-expressing vector, psiCHECK-2. For the luciferase assay, cells were seeded in triplicate in 12-well plates the day before transfection and subsequently transfected with WT or Mut 3′-UTR reporter vector, either in combination with or without the miR-219-5p mimic. Next, cells were harvested and lysed for luciferase activity analysis using the Dual-Luciferase Reporter System (Promega, WI, USA). Three independent experiments were performed.

### Western blot assay

Cells or tissues were lysed, protease inhibitors added to the lysates and centrifuged at 12,000 rpm at 4 °C. Protein concentrations were examined with the BCA kit (Pierce, USA). Next, proteins were separated via 10% SDS-PAGE and transferred to PVDF membranes. The following antibodies were employed for detection of protein expression: HMGA2 (1:500, Santa Cruz, California, USA) or anti-GAPDH (1:2000, Santa Cruz, California, USA) primary antibodies and horseradish peroxidase-conjugated secondary antibody (1:1000, Santa Cruz, California, USA). The ECL chemiluminescent kit (Millipore, MA, USA) was applied to visualize protein bands.

### Transwell assay

Cells were transfected with miR-219-5p mimic or HMGA2 overexpression vector or pretreated with miR-219-5p inhibitor. After 48 h, cells were cultured in serum deprivation medium for 12 h and digested with trypsin before seeding on the top chambers of 24-well transwell culture inserts (Promega). Medium supplemented with 20% serum was used as a chemoattractant in the lower chambers. After 24 h, cells were fixed for 10 min with 4% formalin before staining with 0.005% crystal violet and counted under a phase contrast microscope.

Invasion assays were performed using the BD Bio-Coat Matrigel invasion assay system (BD Biosciences, NJ, USA) according to the manufacturer’s instructions. The non-motile or noninvasive cells were removed while the lower side of the filter was stained with 0.005% crystal violet and counted.

### Tumor growth in vivo

After transfection, 2 × 10^6^ SKOV3 cells were transplanted into nude mice (25–30 g, 6 weeks old, n = 6). Gross tumor volumes were measured with vernier calipers every 5 days from days 5 to 25. Mice were subsequently killed for western blot analysis.

### Statistical analysis

Continuous variables were expressed as mean ± standard deviation (SD). One-way ANOVA was performed for multiple comparisons using GraphPad Prism software, version 5.0 (GraphPad, La Jolla, CA, USA). P-values ≤ 0.05 indicated statistically significant differences.

## Results

### miR-219-5p overexpression suppresses ovarian carcinoma cell proliferation, migration and invasion

**Fig. 1:**
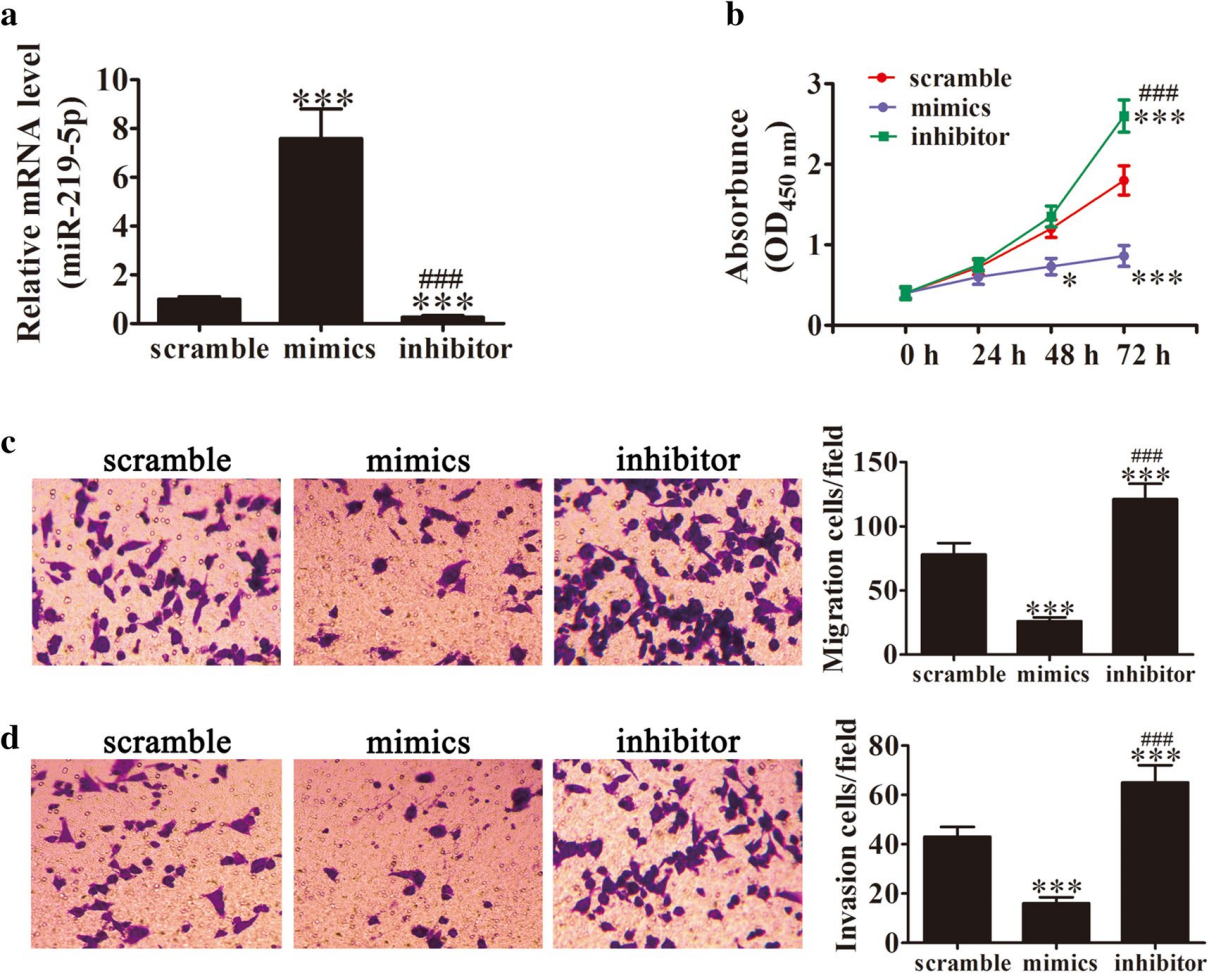
miR-219-5p suppresses ovarian carcinoma cell proliferation, migration and invasion. **a** qRT-PCR showing expression of miR-219-5p in SKOV3 cells after treatment with the miR-219-5p mimic, specific inhibitor or scramble (control) for 48 h. Data are expressed as mean ± SD, n = 5. ^***^P < 0.001 versus scramble. **b** Measurement of SKOV3 cell proliferation with the CCK-8 assay. Data are expressed as mean ± SD, n = 5. *P < 0.05, ***P < 0.001 versus scramble. ^###^P < 0.001 versus the mimic group. **c**, **d** SKOV3 cell migration and invasion determined using the Transwell^®^ assay after treatment with the miR-219-5p mimic, specific inhibitor or scramble. Data are expressed as mean ± SD, n = 5. ***P < 0.001 versus scramble. ^###^P < 0.001 versus the mimic group

To establish the specific role of miR-219-5p, SKOV3 cells were transfected with an miR-219-5p overexpression vector (miR-219-5p mimic) or pretreated with an miR-219-5p inhibitor for 48 h. qRT-PCR results revealed a significant increase in miR-219-5p expression after transfection with the miR-219-5p mimic and conversely, significant suppression of miR-219-5p following treatment with a specific inhibitor, compared with the scramble group (Fig. 1a). Cell proliferation was determined with a cell counting kit using an initial concentration of 1 × 10^4^ cells cultured for different time periods (0, 24, 48, and 72 h). miR-219-5p overexpression led to significant suppression of proliferation of SKOV3 cells from 48 h. In contrast, treatment with the miR-219-5p-specific inhibitor significantly promoted SKOV3 cell proliferation relative to the control group (Fig. 1b). In transwell migration (Fig. 1c) and invasion (Fig. 1d) assays, miR-219-5p overexpression led to significant suppression of migration and invasiveness of SKOV3 cells. Conversely, miR-219-5p inhibitor treatment promoted SKOV3 migration and invasiveness, compared with the scramble group. Our results collectively demonstrate that miR-219-5p exerts inhibitory effects on ovarian cancer cell proliferation, migration and invasion in vitro.

### HMGA2 reverses the miR-219-5p-induced decrease in cell proliferation, migration and invasion

**Fig. 2:**
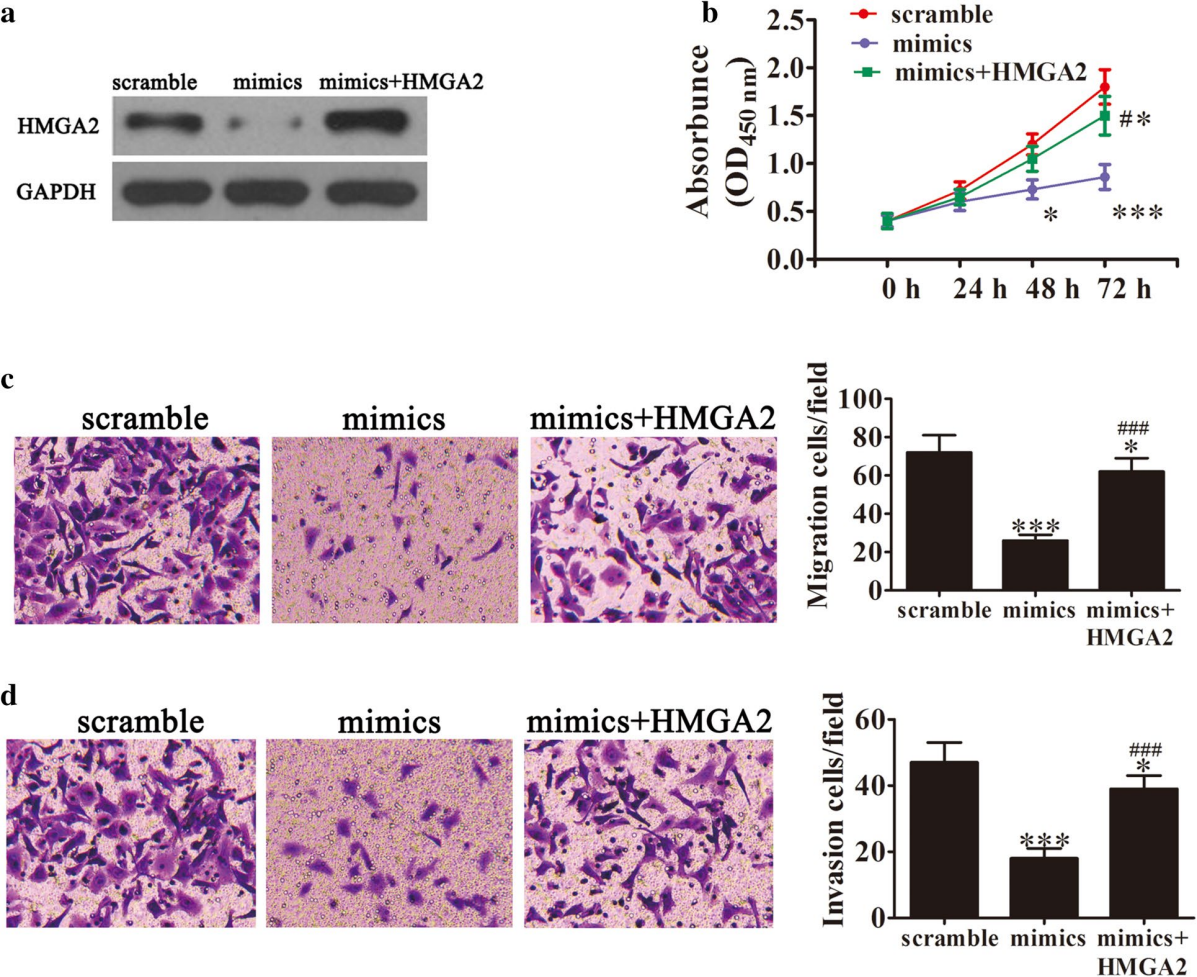
HMGA2 overexpression suppresses miR-219-5p-induced cell proliferation, migration and invasion. **a** Western blot showing HMGA2 expression in SKOV3 cells after treatment with scramble, miR-219-5p mimic or HMGA2 (transfected with HMGA2 overexpression vector). **b** Overexpression of HMGA2 significantly reverses miR-219-5p-induced suppression of SKOV3 cell proliferation. Data are expressed as mean ± SD, n = 5. *P < 0.05, ***P < 0.001 versus scramble. ^#^P < 0.05, ^###^P < 0.001 versus the mimic group. **c**, **d** Cell migration and invasion of SKOV3 determined using the Transwell^®^ assay. Data are expressed as mean ± SD, n = 5. *P < 0.05, ***P < 0.001 versus scramble. ^###^P < 0.001 versus the mimic group

Previous studies have reported the involvement of HMGA2 in different steps of tumorigenesis. High levels of HMGA2 are reported in various cancer types, including ovarian cancer [10, 15]. To determine whether HMGA2 is involved in miR-219-5p activity on tumor cell proliferation, an HMGA2 overexpression vector was constructed and successfully transfected into SKOV3 cells. Western blot results confirmed a significant increase in HMGA2 in SKOV3 cells after transfection with the overexpression vector (Fig. 2a). In previous studies, expression of miR-219-5p led to significant inhibition of SKOV3 cell proliferation. Notably, HMGA2 overexpression reversed the inhibitory effects of miR-219-5p on proliferation of SKOV3 cells (Fig. 2b). Transwell migration and invasion assays further showed that upregulation of HMGA2 significantly reverses miR-219-5p-induced inhibition of migration (Fig. 2c) and invasiveness (Fig. 2d), confirming that HMGA2 is involved in miR-219-5p mediated regulation of ovarian cancer cell growth.

### HMGA2 mRNA is a binding target of miR-219-5p

**Fig. 3:**
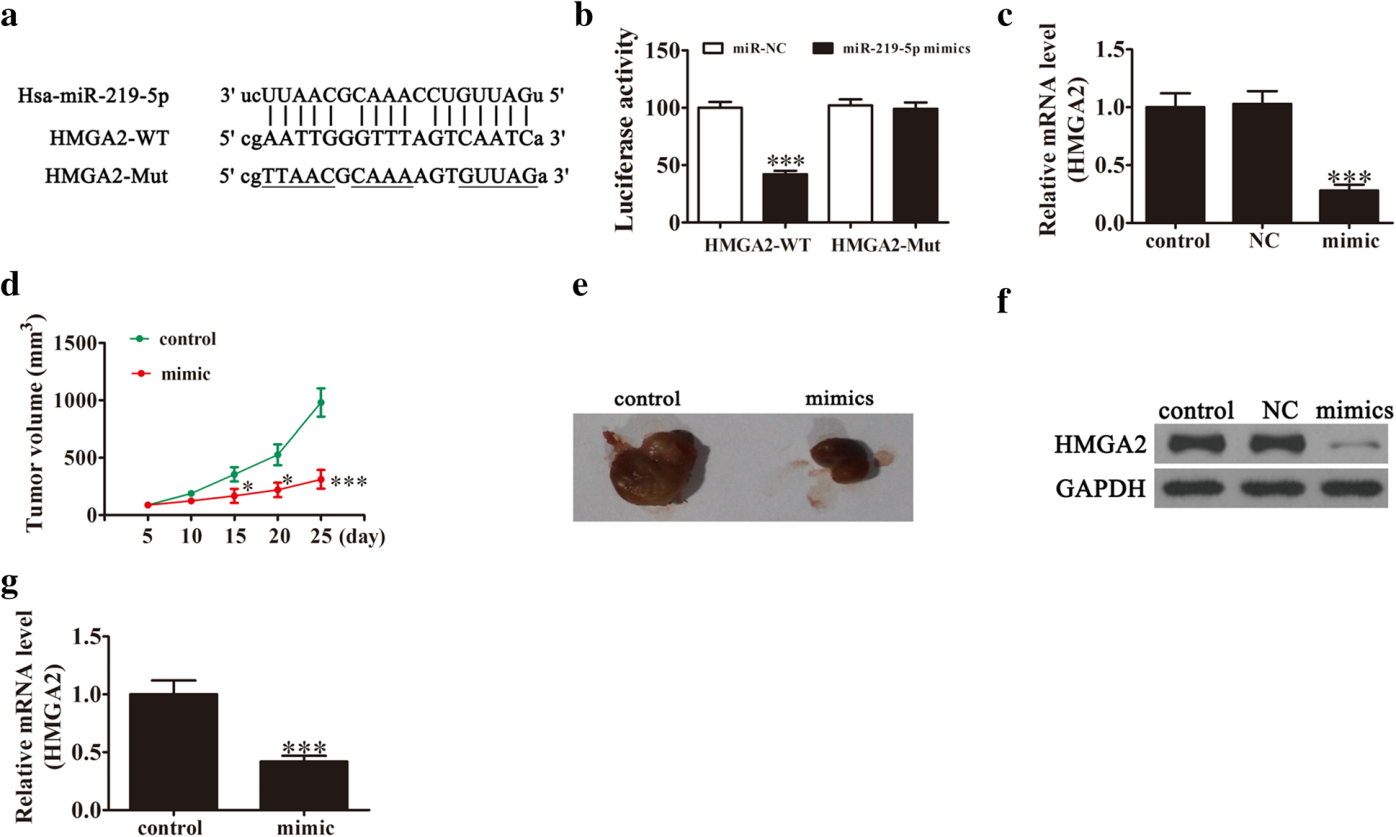
HMGA2 is a functional target of miR-219-5p in SKOV3 cells. **a** miR-219-5p and its predicted binding sequence in WT (wild-type) HMGA2. **b** MiR-219-5p suppresses the luciferase reporter activity of wild-type but has no significant impact on mutant HMGA2. Data are expressed as mean ± SD, n = 5. ***P < 0.001 versus other groups. **c** qRT-PCR showing that miR-219-5p overexpression triggers significant suppression of HMGA2 mRNA. Data are expressed as mean ± SD, n = 5. ***P < 0.001 versus control. **d** Transfection of miR-219-5p decreases tumor growth. Data are expressed as mean ± SD, n = 6. *P < 0.05, ***P < 0.001 versus control. **e** Images of tumor tissues from different groups on day 25. **f**, **g** Western blot and RT-PCR analyses showing that miR-219-5p overexpression triggers a significant decrease in HMGA2 protein and mRNA levels, respectively. Data are expressed as mean ± SD, n = 5. ***P < 0.001 versus control

Two major databases, TargetScan 7.0 and miRanda, were used to search for downstream targets of miR-219-5p, leading to the identification of HMGA2 as a potential target. Data from the luciferase reporter assay showed that the 3′UTR of HMGA2 is a downstream binding target of miR-219-5p (Fig. 3a). Notably, miR-219-5p inhibited luciferase activity in constructs containing wild-type but not mutant 3′UTR sites (Fig. 3b). Our data suggest that miR-219-5p interacts with the 3′UTR of HMGA2 and suppresses post-transcriptional HMGA2 expression. qRT-PCR analyses further confirmed that miR-219-5p overexpression is associated with inhibition of HMGA2 expression (Fig. 3c).

### miR-219-5p inhibits ovarian cancer growth in vivo

In view of the finding that overexpression of miR-219-5p inhibits ovarian cancer cell proliferation in vitro, we further examined its antitumor effects in vivo. Control SKOV3 cells or those stably expressing miR-219-5p were subcutaneously inoculated into nude mice (n = 6 for each group) and the sizes of SKOV3 tumors in mice measured using a caliper every 5 days. Tumor volume was significantly decreased in the group treated with the miR-219-5p mimic, compared with the control groups (Fig. 3d, e). Protein and mRNA expression of HMGA2 in xenograft tumors was determined via western blot (Fig. 3f) and qRT-PCR (Fig. 3g), respectively. HMGA2 was clearly downregulated in xenograft tumors from the miR-219-5p mimic group, compared to xenograft tumors of the control groups. The collective results showed that upregulation of miR-219-5p inhibits ovarian cancer growth in vivo, which may be mediated via regulation of HMGA2 levels.

## Discussion

Tumor development is a complex evolutionary process regulated by both protumor and antitumor elements [16]. Accumulating studies suggest that microRNAs play important roles in tumorigenesis through modulating target gene expression, including cell proliferation, differentiation, apoptosis and metastasis [17]. Abnormal expression and an antitumor role of miR-219-5p in a few cancer types has been documented in the literature. For example, miR-219-5p inhibits the proliferation, migration and invasion of epithelial ovarian cancer cells by targeting the Twist/Wnt/β-catenin signaling pathway [18] and the growth and metastasis of malignant melanoma by targeting BCL-2 [14]. In the current study, miR-219-5p suppressed the proliferation, migration and invasion of ovarian cancer cells through suppression of HMGA2, both in vitro and in vivo.

HMGA2, a small, non-histone, chromatin-associated protein that plays a key role in tumorigenesis, acts as an transcription factor that regulates numerous genes [19]. HMGA2 overexpression has been frequently detected in several malignant neoplasms and inhibition of its expression shown to prevent tumor transformation in several cancer types, including thyroid cell [20], tongue squamous cell carcinoma [21] and breast cancer [22]. Data from this investigation clearly suggest that HMGA2 promotes ovarian cancer development. In our experiments, overexpression of HMGA2 reversed miR-219-5p-induced inhibition of ovarian cancer cell proliferation, migration and invasion. An earlier bi-fluorescein reporter experiment identified HMGA2 as a crucial downstream target of miR-219-5p. However, the precise mechanisms underlying HMGA2-mediated promotion of ovarian cancer cell growth processes require elucidation in future studies.

## Conclusions

In conclusion, miR-219-5p inhibits cell proliferation, invasion and migration of ovarian cancer cells through interactions with the 3′-UTR of HMGA2. This miRNA may provide a novel diagnostic and therapeutic option for patients with ovarian cancer.

## Abbreviations

HMGA2: high-mobility gene group A2
miRNA: microRNA
3′UTR: 3′-untranslated region
FBS: fetal bovine serum
